# Mass-spectrometry-based spatial proteomics data analysis using pRoloc and pRolocdata

**DOI:** 10.1093/bioinformatics/btu013

**Published:** 2014-01-11

**Authors:** Laurent Gatto, Lisa M. Breckels, Samuel Wieczorek, Thomas Burger, Kathryn S. Lilley

**Affiliations:** ^1^Computational Proteomics Unit and ^2^Cambridge Centre for Proteomics, Department of Biochemistry, University of Cambridge, Tennis Court Road, CB2 1QR, Cambridge, UK and ^3^Université Grenoble-Alpes, CEA (iRSTV/BGE), INSERM (U1038), CNRS (FR3425), 38054 Grenoble, France

## Abstract

**Motivation:** Experimental spatial proteomics, i.e. the high-throughput assignment of proteins to sub-cellular compartments based on quantitative proteomics data, promises to shed new light on many biological processes given adequate computational tools.

**Results:** Here we present pRoloc, a complete infrastructure to support and guide the sound analysis of quantitative mass-spectrometry-based spatial proteomics data. It provides functionality for unsupervised and supervised machine learning for data exploration and protein classification and novelty detection to identify new putative sub-cellular clusters. The software builds upon existing infrastructure for data management and data processing.

**Availability:** pRoloc is implemented in the R language and available under an open-source license from the Bioconductor project (http://www.bioconductor.org/). A vignette with a complete tutorial describing data import/export and analysis is included in the package. Test data is available in the companion package pRolocdata.

**Contact:** lg390@cam.ac.uk

## 1 INTRODUCTION

Knowledge of the spatial distribution of proteins is of critical importance to elucidate their role and refine our understanding of cellular processes. Mis-localization of proteins have been associated with cellular dysfunction and disease states (Kau *et al.*, 2004; Laurila *et al.*, 2009; Park *et al.*, 2011), highlighting the importance of localization studies. Spatial or organelle proteomics is the systematic study of the proteins and their sub-cellular localization; these compartments can be organelles, i.e. structures defined by lipid bi-layers, macro-molecular assemblies of proteins and nucleic acids or large protein complexes. Despite technological advances in spatial proteomics experimental designs and progress in mass-spectrometry (Gatto *et al.*, 2010), software support is lacking. To address this, we developed the pRoloc package that provides a wide range of thoroughly documented analysis methodologies. The software includes state-of-the-art statistical machine-learning algorithms and bundles them in a consistent framework, accommodating any experimental designs and quantitation strategies.

## 2 AVAILABLE FUNCTIONALITY

pRoloc makes use of the architecture implemented in the MSnbase package (Gatto and Lilley, 2012) for data storage, feature and sample annotation (meta-data) and data processing, such as scaling, normalization and missing data imputation. We also distribute 16 annotated datasets in the pRolocdata package, which are used for illustration of different pipelines as well as algorithm testing and development. Algorithms for (i) clustering, (ii) novelty detection and (iii) classification are proposed along with visualization functionalities.

### 2.1 Clustering

The unsupervised machine-learning techniques are used, among other aims, as exploration and quality control tools. Several critical factors such as feature-level quantitation values, the extent of missing values and organelle markers can be overlaid on the data clusters as effective data exploration and quality control.

### 2.2 Novelty detection

**Fig. 1.**
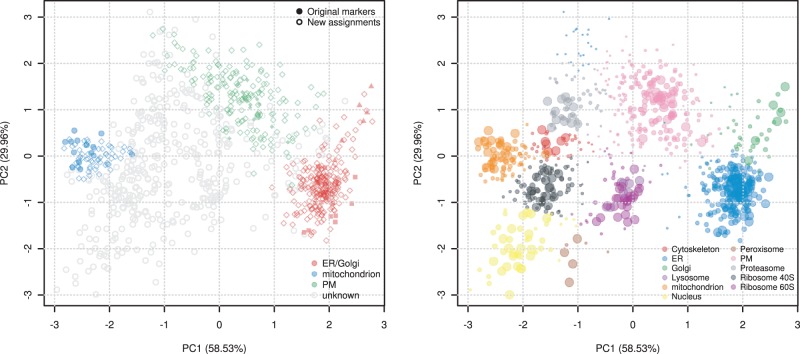
Current state-of-the-art experimental organelle proteomics data analysis with pRoloc. On the left, we replicated the original findings from Tan *et al.* (2009) on *Drosophila* embryos. On the right, we present results of the same data set obtained with pRoloc, utilizing the novelty discovery functionality (new color-coded organelles) and a class-weighted support vector machine (SVM) algorithm with classifier posterior probabilities (point sizes)

An essential step for reliable classification is the availability of well-characterized labeled data, termed ‘marker proteins’. These reliable organelle residents define the set of observed organelles and are used to train a classifier. It is however laborious and extremely difficult to manually define reliable markers for all possible sub-cellular structures. As such, any organelles without any suitable markers will be completely omitted from subsequent classification. pRoloc provides the implementation for the *phenoDisco* novelty detection algorithm (Breckels *et al.*, 2013) that, based on a minimal set of markers and unlabeled data, can be used to effectively detect new putative clusters in the data, beyond those that were initially manually described (Fig. 1).

### 2.3 Classification

Since the development and refinement of spatial proteomics experiments, several classification methods have been used: partial least-square discriminant analysis (Dunkley *et al.*, 2006), SVMs (Trotter *et al.*, 2010), random forest (Ohta *et al.*, 2010), neural networks (Tardif *et al.*, 2012) and naive Bayes (Nikolovski *et al.*, 2012), all available in pRoloc. In addition, other novel algorithms are proposed, such as PerTurbo (Courty *et al.*, 2011). We have compared and contrasted these algorithms using reliable marker sets and demonstrate in the package documentation that the driving factor for good classification is reflected in the intrinsic quality of the data itself, i.e. efficient cellular content separation, accurate quantitation (Jakobsen *et al.*, 2011), etc. illustrating the minor importance of the classification algorithm with respect to thorough data exploration and quality control. While the exact algorithm might not be the major reason for a good analysis, it is essential to guarantee optimal application of the algorithm. A central design decision in the development of the classification schema was to explicitly implement model parameter optimization routines to maximize the generalization power of the results.

## 3 A TYPICAL PIPELINE

A typical pipeline is summarized below using data from *Arabidopsis thaliana* callus (Dunkley *et al.*, 2006). We first load the required packages and example data. The phenoDisco function is then run to identify new putative clusters that, after validation (the pd.markers feature meta-data), can be used for the classification using the SVM algorithm (with a Gaussian kernel). The algorithms parameters are first optimized and then subsequently applied in the actual classification. Finally, the plot2D function is used to generate an annotated scatter plot along the two first principal components (Fig. 1).

library(pRoloc)

library(pRolocdata)

data(dunkley2006)

res <- phenoDisco(dunkley2006)

p <- svmOptimisation(res, fcol="pd.markers")

res <- svmClassification(res, p,

    fcol="pd.markers")

plot2D(res, fcol="svm")

## 4 CONCLUSIONS

The need for statistically sound proteomics data analysis has spawned interest in the proteomics community (Gatto and Christoforou, 2013) for R and Bioconductor (Gentleman *et al.*, 2004). pRoloc is a mature R package that provide users with dedicated data infrastructure, visualization functionality and state-of-the-art machine-learning methodologies, enabling unparalleled insight into experimental spatial proteomics data. It is also a framework to further develop spatial proteomics data analysis and novel pipelines. Multiple organelle proteomics datasets illustrating various and diverse experimental designs are available in pRolocdata. Both packages come with thorough documentation and represent a unique framework for sound and reproducible organelle proteomics data analysis.

*Funding*: European Union 7th Framework Program (PRIME-XS project, grant agreement number 262067); BBSRC Tools and Resources Development Fund (Award BB/K00137X/1); Prospectom project (Mastodons 2012 CNRS challenge).

*Conflict of Interest*: none declared.
